# Upper-extremity deep venous thrombosis and bilateral pulmonary embolism in a patient with COVID-19 under prophylactic anticoagulation: A case report

**DOI:** 10.1016/j.amsu.2022.103485

**Published:** 2022-04-02

**Authors:** Zakariae Belarbi, Falmata Laouan Brem, Noha El Ouafi

**Affiliations:** a Department of Cardiology, Mohammed VI University Hospital, Faculty of Medicine and Pharmacy of Oujda, Mohammed First University, Oujda, Morocco; b Epidemiological Laboratory of Clinical Research and Public Health, Oujda, Morocco

**Keywords:** Case report, COVID-19, Upper-extremity deep venous thrombosis, Brachiocephalic (innominate) vein thrombosis, Pulmonary embolism

## Abstract

The COVID-19 infection induces coagulation dysfunction resulting in an increased incidence of pulmonary embolism (PE) and deep venous thrombosis (DVT), mostly in the lower extremities. While upper-extremity DVT is less frequent than lower-extremity DVT, the thrombosis of internal jugular vein or brachiocephalic (innominate) vein is an uncommon presentation. All the current studies concerning the thrombotic risk linked to hospital COVID-19 indicate that therapeutic anticoagulation does not improve the clinical prognosis in the intensive care unit. Standard prophylactic anticoagulation is therefore recommended. But again, thrombotic complications of COVID-19 infection are still frequently reported nowadays despite anticoagulation therapy, as we can see in this case report. Here we report a rare case of a 50-year-old woman with a previous history of dyslipidemia, admitted for COVID-19 related acute respiratory failure. The patient developed during hospitalization an acute bilateral PE, with upper-extremity DVT including thrombosis of the left brachiocephalic vein extended to the left internal jugular vein, while under prophylactic anticoagulation since hospital admission, leading finally to the patient's death from respiratory failure. At present, the pathophysiology of the hypercoagulable state related to COVID-19 infection is poorly understood. The significant rate of thrombosis despite preventive and therapeutic dosage anticoagulation raises the possibility of a pathophysiology unique to COVID-19. This rare case highlights the importance of thrombotic morbidity and mortality associated with the SARS-CoV-2 epidemic, and the need for further studies to better understand the physiopathology behind the thrombotic state of COVID 19 infection and establish a more efficient way to deal with these complications.

## Highlights

• Thromboembolic complications are commonly associated with COVID-19 infection.
• Thrombosis can occur at different location, requiring minutious examination and close interpretation of imaging.
• Current recommendations indicate prophylactic anticoagulation for the thrombotic risk linked to hospital COVID-19 patients.
• Thromboembolic complications of COVID-19 infection are frequently reported despite anticoagulation therapy.
• The thrombotic state of COVID-19 infection may be related to a unique pathophysiology, wich may require specific therapy that is more effective than standard heparin anticoagulation.

## 1 Introduction

Deep venous thrombosis of the upper-extremities is less frequent than DVT of the lower extremities. The majority of upper-extremities DVT cases are due to secondary causes, including surgery, trauma, immobilization, indwelling catheters, cancer, pregnancy, or oral contraceptives [1,2]. The COVID-19 infection is well known to induce thrombotic disorders, resulting in an increased incidence of PE and DVT, especially in the lower extremities [3,4]. Nevertheless, just a few upper-extremity DVT cases have been previously reported, and it is rarely reported to have all these thrombotic complications despite anticoagulant therapy.

## 2 Case presentation

A 50-year-old woman with a previous history of dyslipidemia, presented to the emergency department for acute respiratory distress after seven days of fever, cough, asthenia, myalgia and headache. The patient had a normal psychological state, and no history of taking drugs or any other suspicious substance. She was stable hemodynamically, with increased breathing at 30 cycles per minute, oxygen saturation at 65% in room air. On physical examination, the patient appears distressed and slightly diaphoretic. Lung auscultation revealed diffuse crackles. Arterial blood gas showed a respiratory alkalosis (pH at 7.52), partial pressure of carbon dioxide (PCO2) at 32,4mmhg, HCO03 at 26,1mmhg, with hypoxemia (partial pressure of oxygen (PO2) at 44mmhg). The SARS-CoV-2 RT-PCR performed on nasopharyngeal swabs was positive. Laboratory findings showed elevated white blood cells (WBC) (17470 elements/mm3), elevated levels of inflammatory markers (CRP at 316mg/L, a high level of ferritin at 818ng/mL, and a high fibrinogen level at 5.9g/L), a high D-Dimer level at (10520ng/mL), and a normal renal function. Based on these findings, the patient was hospitalized in the intensive care unit and received azithromycin 250mg per day, ceftriaxone 2g once a day, and a prophylactic dose of low molecular weight heparin (i.e. 4000 UI once day), with a good drug tolerance.

**Fig. 1:**
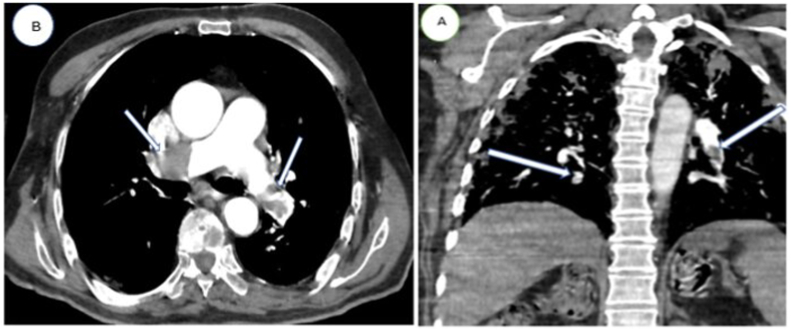
CTPA in coronal (A) and axial (B) lung window showing acute bilateral pulmonary embolism. (white arrows).

**Fig. 2:**
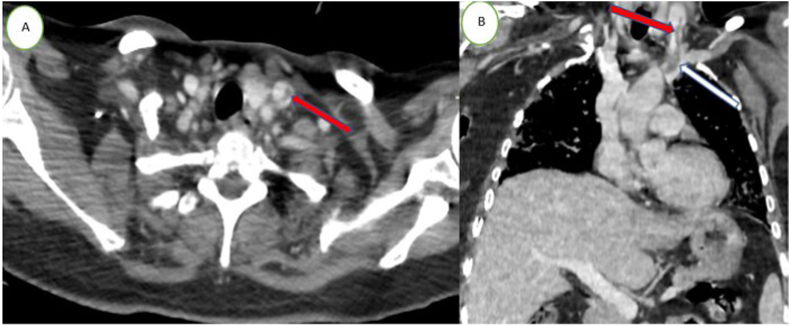
Angiography of chest and neck revealing a thrombosis of left brachiocephalic (innominate) vein extended to the left internal jugular vein.

**Fig. 3:**
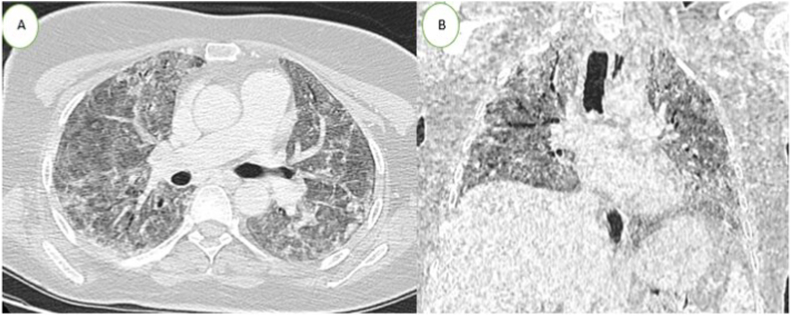
Chest CT scan in axial (A) and coronal (B) lung parenchyma windows revealing COVID-19 pneumonia related signs with more than 75% of lung parenchyma affected.

Five days after admission, the patient reported vague shoulder discomfort, then deteriorated and required intubation with mechanical ventilation. The physical examination showed jugular venous distention. A computed tomography pulmonary angiography (CTPA) showed an acute bilateral pulmonary embolism [Fig. 1 A, B]. Angiography of chest and neck showed a thrombosis of the left brachiocephalic (innominate) vein extended to the left internal jugular vein [Fig. 2 A, B]. Chest CT scan showed COVID-19 pneumonia-related signs with more than 75% of lung parenchyma affected. [Fig. 3] A transthoracic echocardiography was performed, revealing pulmonary arterial hypertension at 48 mmHg estimated by using Tricuspid regurgitation (TR) with peak TR velocity at 3.3 m/s.

We switched to the therapeutic dose of low molecular weight heparin (7000 UI twice a day). But unfortunately, 12 days after admission the patient deceased from respiratory failure despite a marked improvement in the inflammatory assessment. Death occurred from respiratory exhaustion following the pulmonary embolism and the sequelae of the COVID-19 infection.

## 3 Discussion

Upper-extremity DVT, which refers to thrombosis of the internal jugular, brachiocephalic, brachial, subclavian, and/or axillary veins, is rarely reported [5,6]. The annual incidence is 0.4–1 case per 10,000 [7]. Despite anticoagulation, the incidence of venous thromboembolism (VTE) in patients with COVID-19 infection, especially PE and DVT, is interestingly increasing [8]. However, just a few upper extremity DVT cases were reported in patients with COVID-19 infection [[9], [10], [11]]. Furthermore, brachiocephalic vein thrombosis is a sporadic presentation, and to the best of our knowledge, no case has been previously reported in patients infected by the SARS-CoV-2. Our COVID-19-patient developed PE and upper-extremity DVT despite being on prophylactic anticoagulation therapy. This highlights the importance of the prothrombotic state described in patients infected by the SARS-CoV-2.

The clinical signs and symptoms of upper-extremity DVT are nonspecific and may include arm swelling, pain, visible collateral veins at the shoulder girdle or jugular distention. However asymptomatic cases have been reported. Indeed, in our case, there was barely a discreet jugular distention and a vague shoulder discomfort. The diagnosis was made on CT angiography performed for PE suspicion. Besides, for upper-extremity DVT compared to lower-extremity DVT, the diagnostic algorithms using clinical pretest probability and D-dimer have not been validated. Therefore, imaging is needed [2].

Complications of upper extremity DVT include pulmonary embolism and post-thrombotic syndrome, which occur in 6% and 5% of cases, respectively [4]. Besides, there is a risk of recurrences about 9% in this patients [12]. The mortality rate is about 10%–50% 12 months after diagnosis, and this is very high compared with patients who have lower extremity DVT [7]. Our patient also had PE on CTPA in addition to the upper extremity DVT. However, we could not deduce if PE was the complication of the internal vein jugular vein thrombosis or if PE occurred independently.

At present, the pathophysiology of the hypercoagulable condition is poorly understood. The significant rate of thrombosis despite preventive and therapeutic dose anticoagulation raises the possibility of a pathophysiology unique to COVID-19 infection [13,14]. One of the proposed hypotheses is the occurrence of a severely heightened inflammatory response that leads to thrombo-inflammation via mechanisms such as complement activation, cytokine storm and endotheliitis [15,16]. It has also been hypothesized that the virus may itself trigger the coagulation cascade [17]. Current recommendations for anticoagulant therapy indicate a prophylactic dose (i.e. 4000 UI once day) in patients hospitalized in the intensive care unit, since therapeutic dose does not improve the clinical prognosis [18,19]. Nevertheless, we can see that thromboembolic complications can occur despite anticoagulant therapy, and sometimes with vital engagement, as we can see in our patient. The aim of this rare case is to encourage investigation of the pathophysiology of the COVID-19 related coagulopathy, as we support the hypothesis of a physiopathology unique to COVID 19 infection concerning the prothrombotic state, wich could lead to the discovery of new therapeutic perspectives to counter these thromboembolic complications.

## 4 Conclusion

The procoagulant state during COVID 19 is developing as a prominent pathogenic event, given the fulminant increase in thrombotic complications with their consequences on morbidity and mortality in patients infected with COVID 19. Therefore, physicians should maintain high clinical suspicion in screening COVID-19 patients, and approaches to thrombotic complications should be reviewed. This rare case of COVID-19 developing an upper-extremity DVT and pulmonary embolism under prophylactic anticoagulation, highlights the need for further studies to establish a more efficient way to deal with the prothrombotic state of COVID 19 infection.

## Sources of funding

This research did not receive any specific funding.

## Ethical approval

The work has been approved by the appropriate ethical committees related to our institution.

## Registration of research studies

1. Name of the registry:

2. Unique Identifying number or registration ID:

3. Hyperlink to your specific registration (must be publicly accessible and will be checked):

## Consent statement

Written informed consent was obtained from the patient for publication of this case report and accompanying images. A copy of the written consent is available for review by the Editor-in-Chief of this journal on request.

## Author’s contribution

Z. Belarbi, **CONCEPTION, LITERATURE REVIEW**, **ANALYSIS, DATA COLLECTION, WRITING- REVIEW & EDITING**.

F. Laouan Brem: **CONCEPTION, LITERATURE REVIEW**, **ANALYSIS, DATA COLLECTION, WRITING- REVIEW & EDITING**.

N. El Ouafi: **CONCEPTION, METHODOLOGY, SUPERVISION**.

## Guarantor

Belarbi Zakariae

## Provenance and peer review

Not commissioned, externally peer-reviewed.

## Declaration of competing interest

The authors declare non-conflicts of interest.

## References

[1] Khan O., Marmaro A., Cohen D.A. (2021 Aug). A review of upper extremity deep vein thrombosis. Postgrad. Med..

[2] Yuen H.L.A., Tran H., Chunilal S. (2021 Sep). Upper extremity deep vein thrombosis: current knowledge and future directions. Semin. Thromb. Hemost..

[3] Ranucci M., Ballotta A., Di Dedda U., Baryshnikova E., Dei Poli M., Resta M. (2020 Jul). The procoagulant pattern of patients with COVID-19 acute respiratory distress syndrome. J. Thromb. Haemostasis.

[4] Rabaan A.A., Al-Ahmed S.H., Al Mutair A., Alhumaid S., Sule A.A., Tirupathi R. (2021 Jun 1). Immunopathogenesis and immunobiology of SARS-CoV-2. Infez Med.

[5] Czihal M., Hoffmann U. (2011 Jun). Upper extremity deep venous thrombosis. Vasc. Med..

[6] Joffe H.V., Kucher N., Tapson V.F., Goldhaber S.Z. (2004 Sep 21). Deep Vein Thrombosis (DVT) FREE Steering Committee. Upper-extremity deep vein thrombosis: a prospective registry of 592 patients. Circulation.

[7] Kucher N. (2011 Mar 3). Clinical practice. Deep-vein thrombosis of the upper extremities. N. Engl. J. Med..

[8] Paranjpe I., Fuster V., Lala A., Russak A.J., Glicksberg B.S., Levin M.A. (2020 Jul 7). Association of treatment dose anticoagulation with in-hospital survival among hospitalized patients with COVID-19. J. Am. Coll. Cardiol..

[9] Ramalingam S., Arora H., Gunasekaran K., Muruganandam M., Nagaraju S. (2021 Jan 22). Isolated radial vein thrombosis: upper extremity deep vein thrombosis in a patient with COVID-19 infection. Cureus.

[10] Marone E.M., Rinaldi L.F. (2020 Jul). Upsurge of deep venous thrombosis in patients affected by COVID-19: preliminary data and possible explanations. J Vasc Surg Venous Lymphat Disord.

[11] Koritala T., Johnson D.W. (2021 Jul). Internal jugular vein thrombus in coronavirus disease (COVID-19). AJR Am. J. Roentgenol..

[12] Cote L.P., Greenberg S., Caprini J.A., Tafur A., Choi C., Muñoz F.J. (2017 Oct). RIETE investigators. Comparisons between upper and lower extremity deep vein thrombosis: a review of the RIETE registry. Clin. Appl. Thromb. Hemost..

[13] Klok F.A., Kruip M.J.H.A., van der Meer N.J.M., Arbous M.S., Gommers D.A.M.P.J., Kant K.M. (2020 Jul). Incidence of thrombotic complications in critically ill ICU patients with COVID-19. Thromb. Res..

[14] Llitjos J.F., Leclerc M., Chochois C., Monsallier J.M., Ramakers M., Auvray M. (2020 Jul). High incidence of venous thromboembolic events in anticoagulated severe COVID-19 patients. J. Thromb. Haemostasis.

[15] Campbell C.M., Kahwash R. (2020 Jun 2). Will complement inhibition Be the new target in treating COVID-19-related systemic thrombosis?. Circulation.

[16] Varga Z., Flammer A.J., Steiger P., Haberecker M., Andermatt R., Zinkernagel A.S. (2020 May 2). Endothelial cell infection and endotheliitis in COVID-19. Lancet.

[17] Oudkerk M., Büller H.R., Kuijpers D., van Es N., Oudkerk S.F., McLoud T. (2020 Oct). Diagnosis, prevention, and treatment of thromboembolic complications in COVID-19: report of the national institute for public health of The Netherlands. Radiology.

[18] Lopes R.D., de Barros E Silva P.G.M., Furtado R.H.M., Macedo A.V.S., Bronhara B., Damiani L.P. (2021 Jun 12). ACTION Coalition COVID-19 Brazil IV Investigators. Therapeutic versus prophylactic anticoagulation for patients admitted to hospital with COVID-19 and elevated D-dimer concentration (ACTION): an open-label, multicentre, randomised, controlled trial. Lancet.

[19] Inspiration Investigators, Sadeghipour P., Talasaz A.H., Rashidi F., Sharif-Kashani B., Beigmohammadi M.T., Farrokhpour M. (2021 Apr 27). Effect of intermediate-dose vs standard-dose prophylactic anticoagulation on thrombotic events, extracorporeal membrane oxygenation treatment, or mortality among patients with COVID-19 admitted to the intensive care unit: the INSPIRATION randomized clinical trial. JAMA.

